# Dihydropteroate Synthase Gene Mutations in *Pneumocystis* and Sulfa Resistance

**DOI:** 10.3201/eid1010.030994

**Published:** 2004-10

**Authors:** Laurence Huang, Kristina Crothers, Chiara Atzori, Thomas Benfield, Robert Miller, Meja Rabodonirina, Jannik Helweg-Larsen

**Affiliations:** * University of California San Francisco, San Francisco, California, USA;; † Luigi Sacco Hospital, Milan, Italy;; ‡ HS Rigshospitalet, Copenhagen, Denmark;; § University College London, London, United Kingdom;; ¶ Hôspital de la Croiz-Rousse, Lyon, France;; # Hvidovre University Hospital, Copenhagen, Denmark

**Keywords:** perspective, Pneumocystis, Pneumocystis jirovecii, pneumonia, Pneumocystis, dihydropteroate synthase, dihydrofolate reductase, mutation, trimethoprim-sulfamethoxazole, dapsone, drug resistance, microbial

## Abstract

*Pneumocystis* pneumonia (PCP) remains a major cause of illness and death in HIV-infected persons. Sulfa drugs, trimethoprim-sulfamethoxazole (TMP-SMX) and dapsone are mainstays of PCP treatment and prophylaxis. While prophylaxis has reduced the incidence of PCP, its use has raised concerns about development of resistant organisms. The inability to culture human *Pneumocystis*, *Pneumocystis jirovecii*, in a standardized culture system prevents routine susceptibility testing and detection of drug resistance. In other microorganisms, sulfa drug resistance has resulted from specific point mutations in the dihydropteroate synthase (DHPS) gene. Similar mutations have been observed in *P. jirovecii*. Studies have consistently demonstrated a significant association between the use of sulfa drugs for PCP prophylaxis and DHPS gene mutations. Whether these mutations confer resistance to TMP-SMX or dapsone plus trimethoprim for PCP treatment remains unclear. We review studies of DHPS mutations in *P. jirovecii* and summarize the evidence for resistance to sulfamethoxazole and dapsone.

## Abstract 2

We review studies of dihydropteroate synthase gene mutations in *Pneumocystis jirovecii* and summarize the evidence for resistance to sulfamethoxazole and dapsone.

Although decreasing in incidence as a result of combination antiretroviral therapy and effective prophylaxis, *Pneumocystis* pneumonia (PCP), caused by *Pneumocystis jirovecii* (formerly *P. carinii* f. sp. *hominis*), remains the most common AIDS-defining opportunistic infection, as well as the most frequent serious opportunistic infection in HIV-infected persons, in the United States and Europe. Despite the fact that this infection can be prevented, certain patients continue to be at increased risk for PCP. Specifically, PCP frequently signals HIV infection in patients not previously known to be HIV-infected (*1*). Patients who are not receiving regular medical care, as well as those who are not receiving or responding to antiretroviral therapy or prophylaxis, are also at increased risk for PCP (*2*). PCP may also develop in other immunosuppressed populations, such as cancer patients and transplant recipients. Furthermore, PCP remains a leading cause of death among critically ill patients, despite advances in treatment and management (*3*).

The first-line treatment and prophylaxis regimen for PCP is trimethoprim-sulfamethoxazole (TMP-SMX) (*4*). While prophylaxis has been shown to reduce the incidence of PCP, the widespread and long-term use of TMP-SMX in HIV patients has raised concerns regarding the development of resistant organisms. Even short-term exposure to TMP-SMX can be associated with the emergence of TMP-SMX resistance, as has been demonstrated in patients with acute cystitis caused by *Escherichia coli* (*5*). Indeed, an increased number of sulfa-resistant bacteria have been isolated in HIV patients, which coincides with the rise in TMP-SMX prophylaxis for PCP (*6**,**7*). In one study, the prevalence of TMP-SMX–resistant *Staphylococcus aureus* and *Enterobacteriaceae* species isolated in all hospitalized patients increased significantly from <5.5% of isolates before 1986 to 20% in 1995, during which time TMP-SMX prophylaxis was increasing in HIV-infected patients (*6*). In addition, the rise in resistant organisms was significantly more prominent in samples obtained from HIV-infected patients, in whom resistant isolates increased from 6.3% in 1988 to 53% in 1995. Another study found that significantly more TMP-SMX–resistant organisms were isolated from HIV-infected patients who had received TMP-SMX than from patients who had not received TMP-SMX (*7*).

Given the emergence of resistance to TMP-SMX among many bacteria (*8*), concern has focused on the potential development of resistant *Pneumocystis*. Based on animal studies, nearly all of the anti-*Pneumocystis* activity of TMP-SMX is due to sulfamethoxazole (*9*). The development of sulfonamide resistance could result in the failure of sulfamethoxazole as well as dapsone, a sulfone antimicrobial agent also used in the treatment and prophylaxis of PCP. While separate lines of investigation also suggest that *Pneumocystis* may be developing resistance to atovaquone, a second-line PCP treatment and prophylaxis regimen (*10*), we concentrate our review on the evidence for the development of sulfonamide-resistant *Pneumocystis*.

## Mechanisms of Sulfonamide Resistance

Sulfonamides act by interfering with folate synthesis. Since many microorganisms cannot transport folate into cells as mammalian cells can, most prokaryotes and lower eukaryotes must synthesize folates de novo (*11*). Sulfonamides inhibit one of the integral enzymes in folate synthesis, dihydropteroate synthase (DHPS), which catalyzes the condensation of para-aminobenzoic acid and pteridine to form dihydropteroic acid (Figure). Since mammalian cells lack DHPS, sulfonamides can selectively inhibit the growth of various microorganisms. Trimethoprim, part of the fixed combination TMP-SMX, inhibits another of the integral enzymes, the dihydrofolate reductase (DHFR).

**Figure:**
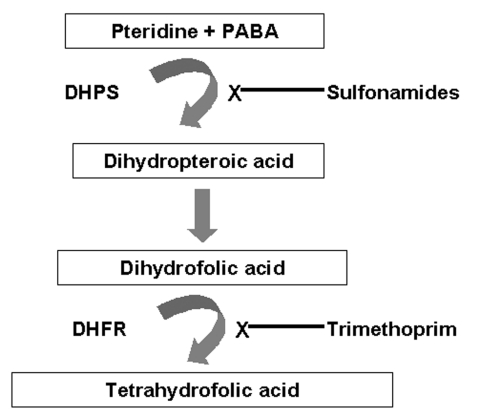
Inhibition of folate synthesis by sulfonamides and trimethoprim. PABA, paraaminobenzoic acid; DHPS, dihydropteroate synthase; DHFR, dihydrofolate reductase.

Resistance to sulfonamides can emerge by means of a number of mechanisms (*12*). In most gram-negative enteric bacteria, sulfonamide resistance is largely plasmid-borne and related to drug-resistant DHPS variants with substantial sequence divergence (*12*). Chromosomal mutations in the DHPS locus—such as point mutations, insertions of duplicate amino acids, or larger sequence alterations as a result of recombination—can also lead to resistance (*8*). In some organisms, several different mechanisms of resistance have been identified in different strains. For example, some strains of *Neisseria meningitidis* have acquired a DHPS gene with 10% sequence divergence, postulated by others to be due to recombination (*12*), whereas other *Neisseria* strains have acquired a chromosomal insertion, resulting in the addition of two amino acids to DHPS (*13*). In other organisms, such as *E. coli* and *Plasmodium falciparum*, nonsynonymous point mutations resulting in amino acid substitutions in DHPS can confer sulfa resistance (*14**,**15*). Furthermore, the accumulation of additional mutations over time can confer increasing levels of sulfa resistance, as has occurred in *P. falciparum* (*16*).

## Dihydropteroate Synthase Mutations in *Pneumocystis*

Similar to other microorganisms, mutations have been identified in the DHPS gene of *Pneumocystis jirovecii*, which has raised the question of whether *P. jirovecii* is developing resistance to sulfonamides. The DHPS gene of *P. jirovecii* has been sequenced and is part of the folic acid synthesis gene or *fas* gene; it encodes a trifunctional protein along with dihydroneopterin aldolase and hydroxymethyldihydropterin pyrophosphokinase (*17*). Sulfa medications appear to exert selective pressure on *Pneumocystis* (*18*), as the DHPS gene is more likely to display mutations in highly conserved regions in patients with PCP who have previously been exposed to sulfa medications (*19**–**25*). These DHPS gene mutations were rarely found in clinical isolates before the early 1990s (*19**,**20**,**22*). Genetic analysis suggests that the mutations arose independently in multiple strains of *Pneumocystis*, which supports the theory that exposure to sulfa medications selects for DHPS gene mutations (*26*). Furthermore, DHPS gene mutations have not been found in other mammalian *Pneumocystis* species that have not been exposed to sulfa medications (*18**,**27*).

Several factors suggest that the mutations observed in *P. jirovecii* may confer resistance to sulfa medications. The region of the DHPS gene in which mutations have been identified is one that is highly conserved among other organisms, including *Plasmodium falciparum*, *Streptococcus pneumoniae*, *E. coli*, and *Bacillus subtilis* (*18*). The most common mutations identified in the *Pneumocystis jirovecii* DHPS are nonsynonymous point mutations, which result in amino acid substitutions at positions 55, 57, or both. Different strains with single or double amino acid substitutions at these positions have been identified (Table 1). Based on homology to the *E. coli* DHPS, these point mutations appear to be in an active site of the enzyme involved in substrate binding; thus, amino acid substitutions in these regions could result in structural changes that could interfere with substrate binding and enzyme activity (*21*). Likewise, similar point mutations in positions equivalent to this site in *Plasmodium falciparum* (*15*) and *Mycobacterium leprae* (*28*) confer sulfa resistance. Other mutations near this site also cause sulfa resistance in *S. pneumoniae* and *P. falciparum* (*18*).

**Table 1:** Amino acid substitutions observed in *Pneumocystis jirovecii*^a^

DHPS genotype	Amino acid position 55	Amino acid position 57
Wild-type		
1	Threonine	Proline
Mutant		
2	Alanine	Proline
3	Threonine	Serine
4 (double mutant)	Alanine	Serine
Mixed		
5	Threonine	Proline/serine
6	Threonine/alanine	Proline/serine
7	Threonine/alanine	Proline
8	Alanine	Proline/serine

^a^DHPS, dihydropteroate synthase.

However, the inability to reliably culture *Pneumocystis jirovecii* in a standardized in vitro culture system prevents the routine susceptibility testing of *Pneumocystis*. The lack of a standardized culture system also hampers research into the development and testing of new antimicrobial agents with anti-*Pneumocystis* activity, which highlights our reliance on TMP-SMX as the current mainstay of therapy. Thus, the clinical significance of these DHPS gene mutations must be inferred from correlating the clinical outcome with the presence of DHPS gene mutations in patients with PCP.

## Association of Sulfamethoxazole and Dapsone with DHPS Gene Mutations

Several studies have consistently demonstrated a significant association between the use of TMP-SMX or dapsone for PCP prophylaxis in HIV-infected persons and the presence of DHPS gene mutations (Table 2) (*19**–**25**,**30*). One study extended these findings to the use of pyrimethamine plus sulfadoxine for PCP prophylaxis (*30*). Another study demonstrated an apparent reversal of the DHPS mutant-to-wild-type ratios after the use of TMP-SMX was restricted (*31*). In total, studies report >700 episodes of PCP, span a period from 1976 to 2001, and include patient data and clinical specimens from multiple cities in several different countries. Unfortunately, these studies used different criteria to define PCP prophylaxis with TMP-SMX or dapsone, which effectively limits attempts at data pooling for more direct and detailed analyses. In addition, most of the studies collected data by abstracting information from patient charts. Thus, these studies were unable to assess whether patients adhered to the prescribed prophylaxis. Nevertheless, seven of the nine studies found that most HIV-infected patients with a diagnosis of PCP who had been prescribed TMP-SMX or dapsone for PCP prophylaxis had *Pneumocystis* that contained DHPS mutations (range 19%–80%, Table 2) (*19**–**24**,**30*). Furthermore, eight of the nine studies reported that PCP patients for whom TMP-SMX or dapsone was prescribed were more likely to have *Pneumocystis* that contained DHPS mutations than were patients for whom these medications were not prescribed (*19**–**25**,**30*).

**Table 2:** Association between sulfonamide or sulfone for PCP prophylaxis and DHPS gene mutations^a^

Author (y) (ref)	PCP cases, no.	Location (time period), country	Prophylaxis^b^ definition (source of information)	DHPS mutations among persons using prophylaxis N (%)	DHPS mutations among persons not using prophylaxis N (%)	p value
Kazanjian (1998) (*19*)	27 (20 HIV-infected)	Ann Arbor, MI (1991–1997), USA		5/7 (71)^c^	2/20 (10)^c^	0.0032
		Indianapolis, IN (1976–1997), USA	At least 1 out of 4 months preceding PCP (chart)	5/7 (71)^d^	2/13 (15)^d^	0.022
Helweg-Larsen (1999) (*20*)	152	Copenhagen (1989–1999), Denmark	Exposure^e^ (chart)	18/29 (62)	13/123 (11)	< 0.0001
			Prophylaxis^f^ (chart)	5/7 (71)^g^	15/125 (12)	0.01
Ma (1999) (*21*)	37 (26 HIV-infected)	Bethesda, MD (1985–1998), USA	Any (chart)	11/14 (79)	2/18 (11)	< 0.001
Kazanjian (2000) (*22*)	97	Denver, CO, Indianapolis, IN, Boston, MA, Detroit, MI (1991–1997), USA	At least 1 out of 4 months preceding PCP (chart)	28/37 (76)	14/60 (23)	< 0.001
Huang (2000) (*23*)	111	Atlanta, GA, Seattle, WA, San Francisco, CA (1996–1999), USA	Ever (interview), Any in the 3 months preceding PCP (chart and interview)	57/71 (80)	19/40 (48)	< 0.001
Visconti (2001) (*24*)	20	Rome (1992–1997), Italy	(Chart)	4/5 (80)	4/15 (27)	0.031
Ma (2002) (*25*)	107	Milan (1994–2001), Italy	Any in the 6 months preceding PCP (chart)	6/31 (19)	3/76 (4)	0.017
Costa (2003) (*29*)	89 (83 HIV-infected)	Lisbon (1994–2001), Portugal	Prophylaxis^h^ Exposure^i^	6/17 (35)	18/72 (25)	0.39
Nahimana (2003) (*30*)	158 (120 HIV-infected)	Lyon (1993–1996), France	Prophylaxis^j^	16/20 (80)	41/138 (30)	< 0.001

^a^PCP, *Pneumocystis* pneumonia; dihydropteroate synthase (DPHS); TMP-SMX, trimethoprim-sulfamethoxazole. ^b^Prophylaxis refers to persons using TMP-SMX or dapsone who met the specific criteria described. ^c^The seven specimens with DHPS gene mutations were from 1995 to 1997. ^d^Analysis restricted to 20 HIV-infected persons. ^e^Exposure, continuous use for at least 1 week at any time after the diagnosis of HIV infection. ^f^Prophylaxis at least 8 weeks preceding PCP. ^g^Analysis restricted to patients receiving TMP-SMX or dapsone prophylaxis versus no prophylaxis. ^h^Prophylaxis, adherence to the same regimen for a minimum of 2 months preceding PCP. ^i^Exposure, use for at least 1 week in the 6 months preceding PCP. ^j^Prophylaxis 3 months preceding PCP. Includes patients who received pyrimethamine/sulfadoxine for PCP prophylaxis.

Of note, in all nine studies, DHPS mutations were observed in PCP patients who were not currently receiving TMP-SMX or dapsone. Whether these patients who failed to meet the defined criteria for TMP-SMX or dapsone prophylaxis had ever received one of these medications for prophylaxis or had received TMP-SMX for a reason other than PCP prophylaxis at some point during their lives was difficult to assess with any degree of confidence. Nevertheless, most of the studies found that only a minority of PCP patients who had not been prescribed TMP-SMX or dapsone for PCP prophylaxis had *Pneumocystis* that contained DHPS mutations. The study that reported the highest proportion (48%) used both chart abstraction and patient interview as sources of clinical information regarding PCP prophylaxis (*23*). This study also used a broad definition of PCP prophylaxis, including patient report of TMP-SMX use for prophylaxis at any time in life. Thus, despite rigorous attempts to document TMP-SMX or dapsone use for PCP prophylaxis and with the broadest definition of prophylaxis applied, nearly half of the patients without TMP-SMX or dapsone use had evidence of DHPS mutations on their clinical PCP specimen. Among the 26 patients with a new diagnosis of HIV infection at the time PCP was diagnosed and who thus had never received PCP prophylaxis, 14 (54%) had *Pneumocystis* that contained DHPS gene mutations. The specific city of residence was also an independent predictor associated with the risk for *Pneumocystis* that contained DHPS gene mutations. Patients who lived in San Francisco were five times more likely, and patients who lived in Seattle were more than three times as likely to have mutant DHPS than patients who resided in Atlanta, even when factors including sulfonamide or dapsone PCP prophylaxis and prior PCP were controlled for. The presence of DHPS mutations in patients without prior TMP-SMX or dapsone use for PCP prophylaxis, the absence of similar mutations in *Pneumocystis* isolated from other mammalian species, and the impact of geography on DHPS genotype have substantial implications for disease transmission (i.e., person-to-person transmission) that are beyond the scope of this review (*32**–**35*).

## Lack of Association of Trimethoprim with DHFR Gene Mutations

Trimethoprim inhibits another of the integral enzymes in folate synthesis, DHFR (Figure). In other microorganisms, point mutations in the DHFR gene are an important mechanism of drug resistance. This finding has led researchers to examine the DHFR gene of *P. jirovecii* to evaluate whether DHFR mutations contribute to TMP-SMX resistance. To date, two studies have failed to demonstrate an association between the use of TMP-SMX for PCP prophylaxis in HIV-infected persons and the presence of DHFR gene mutations (*21**,**36*). In one study, 36 of 37 specimens (from 35 patients, 26 of whom were HIV-infected) demonstrated identical DHFR sequences, with a single specimen showing one synonymous nucleotide change (*21*). In the second study, 16 (59%) of 27 specimens (from 27 patients, 19 of whom were HIV-infected) had DHFR gene mutations, 14 had synonymous changes, and 2 had nonsynonymous changes (*36*). Neither of the two patients whose PCP specimen had nonsynonymous DHFR changes had prior exposure to DHFR inhibitors, yet both patients were treated successfully for PCP with TMP-SMX. In addition, this study aligned the *Pneumocystis* DHFR sequences with those of *E. coli*, *Staphylococcus aureus*, *Streptococcus pneumoniae*, and *Plasmodium falciparum* and reported that the observed nonsynonymous changes in *Pneumocystis* DHFR were not in the highly conserved regions of the enzyme as are the amino acid substitutions that confer resistance to TMP (or pyrimethamine) in these other organisms. Thus, the presence and association of DHPS, but not DHFR, gene mutations with the use of specific PCP prophylaxis regimens argue strongly both for the importance of SMX and dapsone against *Pneumocystis* and the central role of DHPS mutations in the potential development of TMP-SMX or dapsone resistance.

## Clinical Importance of DHPS Gene Mutations in *Pneumocystis jirovecii*

A number of studies have examined the effect of DHPS gene mutations on clinical outcomes such as death, death specifically attributable to PCP, and PCP treatment failure with TMP-SMX or dapsone plus trimethoprim (Table 3) (*19**–**22**,**24**,**25**,**37**–**39*). Whether the presence of DHPS gene mutations confers clinical resistance to TMP-SMX or dapsone plus trimethoprim for PCP treatment remains unclear and requires further study. In a multivariate analysis, Helweg-Larsen and colleagues found that DHPS mutations were an independent predictor associated with increased death rates (*20*). In this study, DHPS mutation was the strongest predictor of death, and patients who had *Pneumocystis* that contained DHPS mutations had a greater than threefold increased risk for death within 3 months compared to patients with the wild-type DHPS, after important mortality cofactors such as age, CD4+/cell count, and arterial oxygen partial pressure (PaO_2_) were controlled for. Whether this increased death rate was due to failure of TMP-SMX for PCP treatment is unclear. In fact, 12 (63%) of 19 PCP patients with *Pneumocystis* that contained DHPS gene mutations responded to PCP treatment with TMP-SMX. Kazanjian and co-workers found that the presence of DHPS mutations was associated with an increased risk for PCP treatment failure with TMP-SMX or dapsone plus trimethoprim (*22*). In univariate analysis, PCP patients who had *Pneumocystis* that contained DHPS gene mutations had a greater than twofold increased risk for treatment failure with one of these regimens, compared to patients with the wild-type DHPS. In this study, treatment failure was defined as worsening of clinical features after 7 days of therapy, failure to improve after 10 days of therapy, or a change in therapy because the treating physician perceives failure. Patients who responded clinically to therapy but who switched therapies because of adverse effects were considered to have been treated successfully. Similar to the findings of Helweg-Larsen, most patients with *Pneumocystis* that contained DHPS gene mutations responded to PCP treatment with TMP-SMX or dapsone plus trimethoprim. Overall, 15 (71%) of 21 PCP patients with *Pneumocystis* that contained DHPS gene mutations responded to PCP treatment with one of these two regimens. In addition, this study found no association between the presence of DHPS mutations and death at 4 weeks. In contrast to these prior two studies, Navin and colleagues found no association between the presence of DHPS mutations and overall number of deaths at 6 weeks, death attributable specifically to PCP, or PCP treatment failure (*39*). Overall, 16 (17%) of 94 PCP patients with DHPS mutations died compared to 9 (25%) of 36 PCP patients with wild-type DHPS (p = 0.30). Similarly, seven patients (7%) with PCP with DHPS mutations died as a result of PCP compared to four patients (11%) with wild-type DHPS. Among the 66 patients with PCP with DHPS mutations who were treated with TMP-SMX, 56 (85%) responded to this treatment. In this study, patients were classified as having been successfully treated if they completed a full course of therapy and responded or if they responded sufficiently to be switched from intravenous to oral therapy and be discharged. Similar to the Kazanjian study, patients who responded clinically to therapy but who switched therapies because of adverse effects were considered to have been treated successfully. This noted TMP-SMX response rate was significantly better than the rate for patients with DHPS mutation who were treated with intravenous pentamidine or clindamycin plus primaquine (14 [50%] of 28) and for patients with the wild-type DHPS who were treated with TMP-SMX (23 [64%] of 36). These results were similar when the analysis was restricted to patients who had been treated for at least 7 days with their initial therapy. Although these three patient groups did not differ in terms of age, CD4-cell count, serum albumin, serum lactate dehydrogenase (LDH), or proportion who required corticosteroids, no multivariate analysis was performed to determine independent predictors associated with death (or PCP treatment failure). Instead, a series of stratified analyses were performed and failed to detect any subsets of PCP patients in whom DHPS mutations were associated with a worse outcome.

**Table 3:** Association between DHPS gene mutations and important clinical outcomes^a^

Author (y) (ref)	PCP cases, no.	DHPS mutations, no.	Increased death rate?	Increased PCP treatment failure?	Comments
Kazanjian (1998) (*19*)	27	7	NA	NA	Both patients with DHPS mutations who were treated with TMP-SMX responded to treatment.
Mei (1998) (*37*)	2	2	NA	NA	2 patients with DHPS mutations were treated with TMP-SMX: 1 did not respond to TMP-SMX (but responded to pentamidine); 1 responded to TMP-SMX.
Helweg-Larsen (1999) (*20*)	144	29	Yes^b^ 3 months	NA	DHPS mutation was an independent predictor associated with increased deaths (OR = 3.1, p = 0.01). 19 patients with DHPS mutations were treated with TMP-SMX: 7 died; 12 (63%) responded and survived.
Ma (1999) (*21*)	37	13	No	NA	
Kazanjian (2000) (*22*)	97	42	No^c^ 4 weeks	Yes^d^	Patients with DHPS mutations were more likely (RR = 2.1, p = 0.01) to fail TMP-SMX or dapsone-containing treatment. Nevertheless, 15 (71%) of 21 patients with DHPS mutations who were treated with TMP-SMX or dapsone-containing regimen responded to treatment.
Takahashi (2000) (*38*)	22	4	NA	Yes	All 4 patients with DHPS mutations who were treated with TMP-SMX did not respond to treatment.
Navin (2001) (*39*)	136	97	No^e^ weeks	No^f^	66 patients with DHPS mutations were treated with TMP-SMX: 56 (85%) responded.
Visconti (2001) (*24*)	20	8	NA	No	1 of 3 patients with DHPS mutations did not respond to TMP-SMX treatment.
Ma (2002) (*25*)	107	9	No^g^ 4 weeks	No	

^a^DHPS, dihydropteroate synthase; PCP, *Pneumocystis* pneumonia; TMP-SMX, trimethoprim-sulfamethoxazole; NA, not available. ^b^Assessed at 3 months. ^c^Assessed at 4 weeks. ^d^Defined as the following: a) deterioration after 7 days of therapy (worsening clinical features or gas exchange parameters—alveolar-arterial O_2_ gradient increase >20 mm Hg from baseline—when available); b) failure of clinical findings to improve after 10 days of therapy; c) physician perception of failure. ^e^Assessed at 6 weeks. Results were similar whether deaths were defined as from all cause or restricted to cases in which PCP was the primary cause of death. ^f^PCP treatment response defined as the following: a) patient completed full course of initial treatment and responded; b) patient responded sufficiently to be discharged on oral medication; c) patient responded to initial treatment but was given another medication because of adverse effects. Results were similar when analysis was restricted to patients who had received at least 7 days of initial PCP treatment. ^g^Assessed at 4 weeks. Deaths included were restricted to cases in which PCP was the primary cause of death.

## Summary and Future Directions

Whether *Pneumocystis* DHPS gene mutations confer clinical resistance to TMP-SMX or dapsone plus trimethoprim for PCP treatment remains unclear. Published studies offer conflicting results. Each study used different definitions for PCP prophylaxis and PCP treatment success or failure, and each examined patient deaths at different time-points, with different methods of statistical analysis. These methodologic differences limit attempts at data pooling for more direct and detailed analyses. The outcome of HIV-infected patients with PCP is a complex issue, with multiple factors affecting death, including those related to the patient (e.g., age), the patient’s overall health status (e.g., serum albumin), the underlying HIV/AIDS (e.g., coexisting opportunistic infections or conditions), and, of course, those specific factors related to PCP (e.g., disease severity, presence of respiratory failure, need for mechanical ventilation, and development of serious complications such as pneumothorax). In each individual report, the overall number of patients studied and the subset of patients who had *Pneumocystis* that contained DHPS mutations and were treated with TMP-SMX or dapsone plus trimethoprim were too small to account for these factors and to detect small differences in outcome that may be related to drug resistance. Furthermore, these and future observational studies that examine DHPS genotype and PCP treatment outcome are complicated by the absence of validated PCP clinical treatment guidelines, practice standards, and definitions of treatment success or failure.

While the declining incidence of PCP in the United States and Europe, as a result of combinations of antiretroviral therapy and PCP prophylaxis, might lessen the enthusiasm for continued study of this issue, brief consideration of a number of factors that warn of a future "perfect storm" suggests that continued study is important. First, most HIV-infected persons worldwide reside in sub-Saharan Africa, Southeast Asia, and Latin America, places where access to antiretroviral therapy and PCP prophylaxis are limited. Second, PCP is increasingly being recognized as an important cause of illness in these regions. In many of these regions, programs to use TMP-SMX as multiopportunistic infection prophylaxis are being implemented, and *Pneumocystis* that contains DHPS mutations can be expected. Next, the treatment options for PCP in these regions are often limited to TMP-SMX, since regimens such as pentamidine, clindamycin plus primaquine, trimetrexate, and atovaquone are unavailable. The existence of TMP-SMX–resistant *Pneumocystis* in these regions, combined with the general absence of invasive diagnostic procedures (e.g., bronchoscopy that might establish an earlier diagnosis of PCP when the outcome is better) and intensive care facilities (e.g., mechanical ventilation that might support patients until PCP treatment can be effective), stresses the importance of further study (*40*).

## References

[1] Kaplan JE, Hanson D, Dworkin MS, Frederick T, Bertolli J, Lindegren ML, Epidemiology of human immunodeficiency virus-associated opportunistic infections in the United States in the era of highly active antiretroviral therapy. Clin Infect Dis. 2000;30(Suppl 1):S5–14. 10.1086/313843 10770911

[2] Lundberg BE, Davidson AJ, Burman WJ. Epidemiology of *Pneumocystis carinii* pneumonia in an era of effective prophylaxis: the relative contribution of non-adherence and drug failure. AIDS. 2000;14:2559–66. 10.1097/00002030-200011100-00019 11101068

[3] Morris A, Wachter RM, Luce J, Turner J, Huang L. Improved survival with highly active antiretroviral therapy in HIV-infected patients with severe *Pneumocystis carinii* pneumonia. AIDS. 2003;17:73–80. 10.1097/00002030-200301030-00010 12478071

[4] Kaplan JE, Masur H, Holmes KK. Guidelines for preventing opportunistic infections among HIV-infected persons–2002. Recommendations of the U.S. Public Health Service and the Infectious Diseases Society of America. MMWR Recomm Rep. 2002;51(RR-8):1–52. 12081007

[5] Brown PD, Freeman A, Foxman B. Prevalence and predictors of trimethoprim-sulfamethoxazole resistance among uropathogenic *Escherichia coli* isolates in Michigan. Clin Infect Dis. 2002;34:1061–6. 10.1086/339491 11914994

[6] Martin JN, Rose DA, Hadley WK, Perdreau-Remington F, Lam PK, Gerberding JL. Emergence of trimethoprim-sulfamethoxazole resistance in the AIDS era. J Infect Dis. 1999;180:1809–18. 10.1086/315132 10558935

[7] Wininger DA, Fass RJ. Impact of trimethoprim-sulfamethoxazole prophylaxis on etiology and susceptibilities of pathogens causing human immunodeficiency virus-associated bacteremia. Antimicrob Agents Chemother. 2002;46:594–7. 10.1128/AAC.46.2.594-597.2002 11796387 PMC127034

[8] Huovinen P. Resistance to trimethoprim-sulfamethoxazole. Clin Infect Dis. 2001;32:1608–14. 10.1086/320532 11340533

[9] Walzer PD, Foy J, Steele P, Kim CK, White M, Klein RS, Activities of antifolate, antiviral, and other drugs in an immunosuppressed rat model of *Pneumocystis carinii* pneumonia. Antimicrob Agents Chemother. 1992;36:1935–42. 1416884 10.1128/aac.36.9.1935 PMC192212

[10] Kazanjian P, Armstrong W, Hossler PA, Lee CH, Huang L, Beard CB, *Pneumocystis carinii* cytochrome b mutations are associated with atovaquone exposure in patients with AIDS. J Infect Dis. 2001;183:819–22. 10.1086/318835 11181161

[11] Skold O. Sulfonamide resistance: mechanisms and trends. Drug Resist Updat. 2000;3:155–60. 10.1054/drup.2000.0146 11498380

[12] Huovinen P, Sundstrom L, Swedberg G, Skold O. Trimethoprim and sulfonamide resistance. Antimicrob Agents Chemother. 1995;39:279–89. 7726483 10.1128/aac.39.2.279 PMC162528

[13] Qvarnstrom Y, Swedberg G. Additive effects of a two-amino-acid insertion and a single-amino-acid substitution in dihydropteroate synthase for the development of sulphonamide-resistant *Neisseria meningitidis.* Microbiology. 2000;146:1151–6. 10832642 10.1099/00221287-146-5-1151

[14] Dallas WS, Gowen JE, Ray PH, Cox MJ, Dev IK. Cloning, sequencing, and enhanced expression of the dihydropteroate synthase gene of *Escherichia coli* MC4100. J Bacteriol. 1992;174:5961–70. 1522070 10.1128/jb.174.18.5961-5970.1992 PMC207134

[15] Brooks DR, Wang P, Read M, Watkins WM, Sims PF, Hyde JE. Sequence variation of the hydroxymethyldihydropterin pyrophosphokinase: dihydropteroate synthase gene in lines of the human malaria parasite, *Plasmodium falciparum*, with differing resistance to sulfadoxine. Eur J Biochem. 1994;224:397–405. 10.1111/j.1432-1033.1994.00397.x 7925353

[16] Nzila AM, Mberu EK, Sulo J, Dayo H, Winstanley PA, Sibley CH, Towards an understanding of the mechanism of pyrimethamine-sulfadoxine resistance in *Plasmodium falciparum*: genotyping of dihydrofolate reductase and dihydropteroate synthase of Kenyan parasites. Antimicrob Agents Chemother. 2000;44:991–6. 10.1128/AAC.44.4.991-996.2000 10722502 PMC89803

[17] Volpe F, Dyer M, Scaife JG, Darby G, Stammers DK, Delves CJ. The multifunctional folic acid synthesis *fas* gene of *Pneumocystis carinii* appears to encode dihydropteroate synthase and hydroxymethyldihydropterin pyrophosphokinase. Gene. 1992;112:213–8. 10.1016/0378-1119(92)90378-3 1313386

[18] Lane BR, Ast JC, Hossler PA, Mindell DP, Bartlett MS, Smith JW, Dihydropteroate synthase polymorphisms in *Pneumocystis carinii.* J Infect Dis. 1997;175:482–5. 10.1093/infdis/175.2.482 9203679

[19] Kazanjian P, Locke AB, Hossler PA, Lane BR, Bartlett MS, Smith JW, *Pneumocystis carinii* mutations associated with sulfa and sulfone prophylaxis failures in AIDS patients. AIDS. 1998;12:873–8. 10.1097/00002030-199808000-00009 9631140

[20] Helweg-Larsen J, Benfield TL, Eugen-Olsen J, Lundgren JD, Lundgren B. Effects of mutations in *Pneumocystis carinii* dihydropteroate synthase gene on outcome of AIDS-associated *P. carinii* pneumonia. Lancet. 1999;354:1347–51. 10.1016/S0140-6736(99)03320-6 10533864

[21] Ma L, Borio L, Masur H, Kovacs JA. *Pneumocystis carinii* dihydropteroate synthase but not dihydrofolate reductase gene mutations correlate with prior trimethoprim-sulfamethoxazole or dapsone use. J Infect Dis. 1999;180:1969–78. 10.1086/315148 10558954

[22] Kazanjian P, Armstrong W, Hossler PA, Burman W, Richardson J, Lee CH, *Pneumocystis carinii* mutations are associated with duration of sulfa or sulfone prophylaxis exposure in AIDS patients. J Infect Dis. 2000;182:551–7. 10.1086/315719 10915088

[23] Huang L, Beard CB, Creasman J, Levy D, Duchin JS, Lee S, Sulfa or sulfone prophylaxis and geographic region predict mutations in the *Pneumocystis carinii* dihydropteroate synthase gene. J Infect Dis. 2000;182:1192–8. 10.1086/315824 10979917

[24] Visconti E, Ortona E, Mencarini P, Margutti P, Marinaci S, Zolfo M, Mutations in dihydropteroate synthase gene of *Pneumocystis carinii* in HIV patients with *Pneumocystis carinii* pneumonia. Int J Antimicrob Agents. 2001;18:547–51. 10.1016/S0924-8579(01)00460-5 11738342

[25] Ma L, Kovacs JA, Cargnel A, Valerio A, Fantoni G, Atzori C. Mutations in the dihydropteroate synthase gene of human-derived *Pneumocystis carinii* isolates from Italy are infrequent but correlate with prior sulfa prophylaxis. J Infect Dis. 2002;185:1530–2. 10.1086/340220 11992293

[26] Ma L, Kovacs JA. Genetic analysis of multiple loci suggests that mutations in the *Pneumocystis carinii* f. sp. hominis dihydropteroate synthase gene arose independently in multiple strains. Antimicrob Agents Chemother. 2001;45:3213–5. 10.1128/AAC.45.11.3213-3215.2001 11600382 PMC90808

[27] Demanche C, Guillot J, Berthelemy M, Petitt T, Roux P, Wakefield AE. Absence of mutations associated with sulfa resistance in *Pneumocystis carinii* dihydropteroate synthase gene from non-human primates. Med Mycol. 2002;40:315–8. 12146763 10.1080/mmy.40.3.315.318

[28] Kai M, Matsuoka M, Nakata N, Maeda S, Gidoh M, Maeda Y, Diaminodiphenylsulfone resistance of *Mycobacterium leprae* due to mutations in the dihydropteroate synthase gene. FEMS Microbiol Lett. 1999;177:231–5. 10.1111/j.1574-6968.1999.tb13737.x 10474189

[29] Costa MC, Helweg-Larsen J, Lundgren B, Antunes F, Matos O. Mutations in the dihydropteroate synthase gene of *Pneumocystis jirovecii* isolates from Portuguese patients with *Pneumocystis* pneumonia. Int J Antimicrob Agents. 2003;22:516–20. 10.1016/S0924-8579(03)00122-5 14602371

[30] Nahimana A, Rabodonirina M, Zanetti G, Meneau I, Francioli P, Bille J, Association between a specific *Pneumocystis jiroveci* dihydropteroate synthase mutation and failure of pyrimethamine/sulfadoxine prophylaxis in human immunodeficiency virus-positive and -negative patients. J Infect Dis. 2003;188:1017–23. 10.1086/378239 14513422

[31] Miller RF, Lindley AR, Ambrose HE, Malin AS, Wakefield AE. Genotypes of *Pneumocystis jiroveci* isolates obtained in Harare, Zimbabwe, and London, United Kingdom. Antimicrob Agents Chemother. 2003;47:3979–81. 10.1128/AAC.47.12.3979-3981.2003 14638515 PMC296206

[32] Beard CB, Carter JL, Keely SP, Huang L, Pieniazek NJ, Moura IN, Genetic variation in *Pneumocystis carinii* isolates from different geographic regions: implications for transmission. Emerg Infect Dis. 2000;6:265–72. 10.3201/eid0603.000306 10827116 PMC2640877

[33] Huang L, Friedly J, Morris AM, Carter JL, Turner JR, Merrifield C, *Pneumocystis carinii* dihydropteroate synthase genotypes in HIV-infected persons residing in San Francisco: possible implications for disease transmission. J Eukaryot Microbiol. 2001;48:137S–8. 10.1111/j.1550-7408.2001.tb00487.x 11906028

[34] Latouche S, Lacube P, Maury E, Bolognini J, Develous M, Girard PM, *Pneumocystis jirovecii* dihydropteroate synthase genotypes in French patients with pneumocystosis: a 1998–2001 prospective study. Med Mycol. 2003;41:533–7. 10.1080/13693780310001615394 14725329

[35] Crothers K, Huang L, Morris A, Fox M, Groner G, Turner JR, *Pneumocystis* dihydropteroate synthase mutations in patients with *Pneumocystis pneumonia* who are newly diagnosed with HIV infection. J Eukaryot Microbiol. 2003;50(Suppl):609–10. 10.1111/j.1550-7408.2003.tb00648.x 14736181

[36] Takahashi T, Endo T, Nakamura T, Sakashitat H, Kimurat K, Ohnishit K, Dihydrofolate reductase gene polymorphisms in *Pneumocystis carinii* f. sp. hominis in Japan. J Med Microbiol. 2002;51:510–5. 12018659 10.1099/0022-1317-51-6-510

[37] Mei Q, Gurunathan S, Masur H, Kovacs JA. Failure of co-trimoxazole in *Pneumocystis carinii* infection and mutations in dihydropteroate synthase gene. Lancet. 1998;351:1631–2. 10.1016/S0140-6736(05)77687-X 9620722

[38] Takahashi T, Hosoya N, Endo T, Nakamura T, Sakashita H, Kimura K, Relationship between mutations in dihydropteroate synthase of *Pneumocystis carinii* f. sp. hominis isolates in Japan and resistance to sulfonamide therapy. J Clin Microbiol. 2000;38:3161–4. 10970350 10.1128/jcm.38.9.3161-3164.2000 PMC87344

[39] Navin TR, Beard CB, Huang L, del Rio C, Lee S, Pieniazek NJ, Effect of mutations in *Pneumocystis carinii* dihydropteroate synthase gene on outcome of *P. carinii* pneumonia in patients with HIV-1: a prospective study. Lancet. 2001;358:545–9. 10.1016/S0140-6736(01)05705-1 11520525

[40] Huang L, Morris AM, Beard CB. *Pneumocystis carinii* dihydropteroate synthase mutations and treatment with sulfa or sulfone regimens: a proposal for standardized definitions for clinical evaluation. J Eukaryot Microbiol. 2001;48:180S–1. 10.1111/j.1550-7408.2001.tb00510.x 11906054

